# Seminal Redox Improvement and Sperm Proteome Remodeling After Deprox-HP Nutraceutical Supplementation in Male Accessory Gland Inflammation: A Pilot Study

**DOI:** 10.3390/ijms27052498

**Published:** 2026-03-09

**Authors:** Fiorella Di Nicuolo, Alessandro Oliva, Emanuele Pierpaolo Vodola, Michela Cicchinelli, Federica Iavarone, Carla Di Nardo, Edoardo Vergani, Paola Girardi, Francesca Mancini, Alfredo Pontecorvi, Andrea Urbani, Silvia Baroni, Domenico Milardi, Emanuela Teveroni

**Affiliations:** 1 Unit of Chemistry, Biochemistry and Molecular Biology, “A. Gemelli” Hospital Foundation IRCCS, Largo A. Gemelli, 8, 00168 Rome, Italy; fiorella.dinicuolo@policlinicogemelli.it (F.D.N.); michela.cicchinelli@unicatt.it (M.C.); federica.iavarone@unicatt.it (F.I.); francesca.mancini@policlinicogemelli.it (F.M.); andrea.urbani@policlinicogemelli.it (A.U.); silvia.baroni@policlinicogemelli.it (S.B.); emanuela.teveroni@guest.policlinicogemelli.it (E.T.); 2 International Scientific Institute Paul VI, Università Cattolica del Sacro Cuore, Fondazione Policlinico Universitario “A. Gemelli” IRCCS, 00168 Rome, Italy; 3 Complex Operative Unit of Internal Medicine, Endocrinology and Diabetology, Department of Translational Medicine and Surgery, Fondazione Policlinico Universitario “A. Gemelli” IRCCS, 00168 Rome, Italy; alessandro.oliva@guest.policlinicogemelli.it (A.O.); emanuele.vodola@gmail.com (E.P.V.); carla.dinardo@guest.policlinicogemelli.it (C.D.N.); edoardo.vergani@guest.policlinicogemelli.it (E.V.); paola.girardi@guest.policlinicogemelli.it (P.G.); alfredo.pontecorvi@policlinicogemelli.it (A.P.); 4 Department of Medicine and Translational Surgery, General Pathology Section, Università Cattolica del Sacro Cuore, 00168 Rome, Italy; 5 Department of Basic Biotechnological Sciences, Intensive and Perioperative Clinics, Università Cattolica del Sacro Cuore, 00168 Rome, Italy

**Keywords:** MAGI, sperm motility, sperm proteomics, lipid peroxidation, Deprox-HP, ROS

## Abstract

Male accessory gland inflammation (MAGI) can impair male fertility through inflammation-driven oxidative stress and direct sperm damage; nutraceutical approaches may be useful when antibiotics are not indicated. Here, we evaluated a 3-month treatment with a Graminex™-based dietary supplement (Deprox-HP) in twenty MAGI patients integrating conventional semen analysis and oxidative stress assessment with sperm proteomics before and after therapy. After treatment, total and progressive sperm motility increased significantly, whereas sperm concentration and sperm morphology showed a non-significant upward trend. Sperm lipid peroxidation decreased markedly, while the antioxidant capacity showed a non-significant increase. Analysis of the sperm proteome demonstrated a clear PRE–POST clustering, consistent with treatment-associated remodeling. POST samples showed upregulation of proteins linked to sperm motility, redox homeostasis, mitochondrial metabolism and membrane remodeling. Two pregnancies occurred during the treatment period; in both cases, lipid peroxidation decreased along with an increase of morphologically typical spermatozoa, and sperm proteomics showed a concordant post-treatment shift enriched in flagellar and mitochondrial respiratory/redox compartments. Moreover, we found a selective enrichment POST treatment in these two patients of TEX50, a crucial protein involved in acrosome/head-stability during epididymal transit. Overall, Deprox-HP was associated with reduced oxidative membrane damage and a coordinated sperm proteomic shift consistent with improved motility.

## 1. Introduction

Male accessory gland inflammation/infection (MAGI) prevalence among infertile men has been reported to range from ~2% to 18%, largely depending on the diagnostic criteria applied, including ultrasound-based definitions [1,2,3]. MAGI are currently classified as uncomplicated (prostatitis) and complicated forms, when inflammation also involves the seminal vesicles and epididymis (prostatovesiculitis, prostato-vesiculo-epididymitis), and are further categorized as unilateral or bilateral depending on the anatomical extent [4]. Inflammatory involvement of the accessory glands may compromise male fertility through four main, non-exclusive mechanisms: (i) altered glandular secretions with consequent changes in seminal plasma composition (including pH, viscosity, enzymatic activity, and the availability of key factors supporting sperm function), (ii) partial or complete obstruction of the seminal tract with impaired semen emission and accessory gland emptying, (iii) direct sperm injury mediated by leukocytes, bacterial products and/or inflammatory mediators (affecting membrane integrity, mitochondrial function and DNA/chromatin quality), and (iv) increased production of reactive oxygen species (ROS) and inflammatory cytokines that can disrupt redox balance and interfere with motility-related signaling, capacitation, and the acrosome reaction [4].

MAGI-related direct sperm damage encompasses both conventional semen parameters (concentration, motility, morphology) and biofunctional alterations, including mitochondrial membrane potential and viability consistent with apoptosis-related injury [2,5]. MAGI has also been associated with increased sperm chromatin fragmentation, which correlates with poorer reproductive outcomes such as recurrent pregnancy loss [6]. Lipid peroxidation represents another key feature of sperm impairment in MAGI, arising within an inflammatory microenvironment characterized by ROS excess and cytokine production such as IL-1β, IL-6 and IL-8 [7]. Lipid peroxidation refers to ROS-driven oxidative degradation of membrane lipids (particularly polyunsaturated fatty acids), generating reactive lipid-derived products and compromising membrane structure and function; in spermatozoa, membrane lipid damage compromises motility, the acrosome reaction, and sperm–oocyte fusion ability [7,8].

Physiologically, male reproduction relies on a balance between ROS generation and antioxidant buffering. At low levels, ROS serve as signaling mediators in sperm maturation, hyperactivation, capacitation, acrosome reaction, and sperm–oocyte fusion [9]. Endogenous ROS mainly derive from sperm mitochondrial activity, whereas leukocyte infiltration in semen provides an additional intrinsic source, particularly during infection/inflammation [10]. Exogenous ROS contributors include unhealthy lifestyle factors (alcohol, obesity, nutritional deficiencies), smoking, environmental pollutants, and radiation exposure [11]. Antioxidant defenses are largely provided by seminal plasma, as sperm progressively lose cytoplasm during spermatogenesis and thus retain limited intracellular antioxidant stores [12]. In particular, the enzymatic antioxidant defenses found in seminal plasma include superoxide dismutase (SOD), catalase (CAT), and glutathione peroxidase (GPx), whereas non-enzymatic antioxidants include vitamins A and C, carnitine, glutathione (GSH), and pyruvate [12]. When ROS burden exceeds antioxidant capacity, oxidative stress ensues and can impair fertility via the mechanisms described above.

Common therapy for MAGI includes targeted antibiotics, although these are not indicated in abacterial forms or persistent/subacute inflammatory phenotypes. In such settings—especially with negative semen cultures—nutraceuticals with anti-inflammatory/antioxidant activity have attracted interest. Pollen-derived preparations have been proposed as candidate nutraceuticals given their complex composition. Graminex™ is a standardized mixture of pollen extracts (rye, corn, timothy grass), used alone or with vitamins addition; among its best-studied constituents, the phenolic metabolite carvacrol has been reported to inhibit Nuclear Factor kappa-light-chain-enhancer of activated B cells (NF-κB) signaling and support anti-inflammatory effects [13].

In light of this background, we evaluated a 3-month treatment with a Graminex™-based dietary supplement (Deprox-HP) in patients diagnosed with complicated MAGI enrolled in a clinical study (ID5878), assessing oxidative stress in seminal plasma and semen quality as the main clinical endpoint. We also profiled the sperm proteome pre- and post-therapy to identify treatment-associated molecular changes and proteomic signatures consistent with inflammation-driven direct gamete injury, with the goal of highlighting novel diagnostic and prognostic factors for MAGI clinical management.

## 2. Results

### 2.1. Semen Quality and Oxidative Stress Evaluation After 3-Month Deprox-HP Treatment

**Figure 1:**
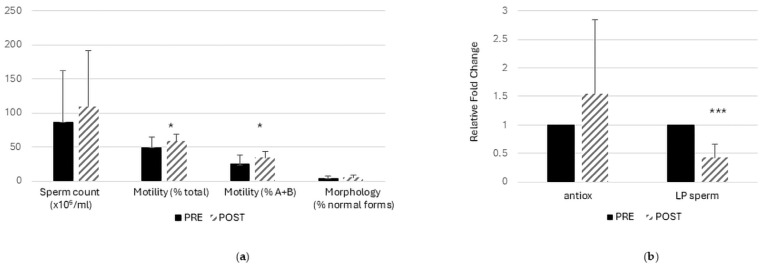
**Deprox-HP supplementation is associated with functional improvement in semen parameters**. (**a**) Conventional semen parameters in twenty MAGI patients, including sperm concentration (×10^6^/mL), total motility (%), progressive motility (A + B, %), and morphologically normal forms (%). (**b**) Seminal antioxidant capacity (antiox) and sperm lipid peroxidation (LP_sperm); values relative to the PRE condition are arbitrarily set to 1 (PRE = 1), and one sample *t*-test was applied as statistical test. Bars represent mean ± SD (n = 20). Statistical significance is indicated as * *p* < 0.05 and *** *p* < 0.001 for PRE vs. POST comparisons.

After 3 months of nutraceutical administration, semen analysis showed a selective improvement in motility (Figure 1a). Both total and progressive motility (A + B) increased significantly in the POST condition compared with PRE, whereas sperm concentration displayed an upward trend without reaching statistical significance (Figure 1a). The percentage of morphologically normal spermatozoa did not materially change over the treatment period (Figure 1a).

Consistent with an antioxidant-driven effect, the seminal antioxidant capacity showed an increasing trend after treatment, although variability was high (Figure 1b). In contrast, sperm lipid peroxidation was markedly reduced in POST versus PRE, reaching strong statistical significance (*p* < 0.001 ***, Figure 1b). Overall, these findings suggest that Deprox-HP treatment is associated with a reduction in lipid peroxidation—although assessed using a single assay—as an indicator of oxidative membrane damage, together with a measurable improvement in sperm motility as a functional semen endpoint.

### 2.2. Proteomic Remodeling of Purified Spermatozoa After Deprox-HP Treatment

**Figure 2:**
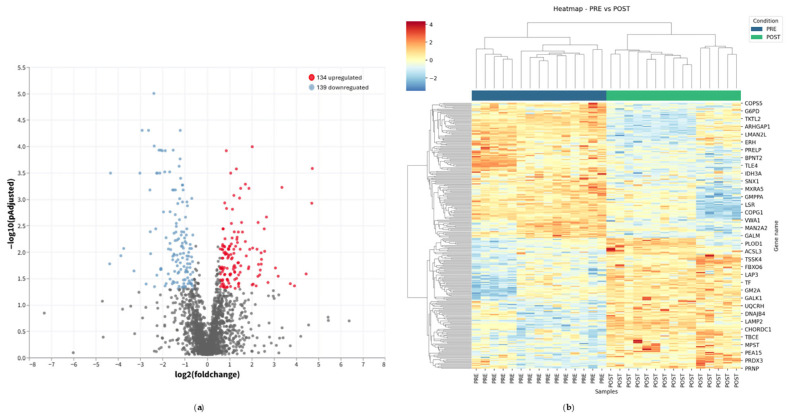
**Differential sperm proteome remodeling after Deprox-HP treatment.** (**a**) Volcano plot of the sperm proteome comparing POST versus PRE samples (n = 15). Each dot represents one quantified protein and is plotted as log2 (POST/PRE fold change) on the x-axis and −log10 (adjusted *p* value) on the y-axis, providing a global view of the 273 differentially expressed proteins across the dataset (up- and downregulated). Blue dots represent downregulated proteins (139), and red dots the upregulated ones (134). (**b**) Hierarchical clustering heatmap of the most significantly differentially abundant proteins between POST and PRE conditions. Rows represent proteins and columns represent individual samples; color intensity indicates scaled relative abundance. The top annotation bar denotes sample condition (PRE vs. POST).

Following Deprox-HP administration, differential expression analysis of the sperm proteome revealed a structured PRE–POST remodeling. Supplementary File S1 lists all the identified proteins, while Supplementary File S2 reports the sequence coverage and peptide counts for all proteins included, ranked by coverage from highest to lowest (Supplementary File S2). The volcano plot (Figure 2a) provides an overview of the entire set of 273 differentially expressed proteins by displaying, for each quantified protein, the magnitude of logarithmic fold-change (POST/PRE) together with its statistical support. This representation highlights the global distribution of post-treatment changes, reporting both up- (134 proteins) and downregulated proteins (139) across the dataset. In accordance with this, hierarchical clustering of differentially expressed proteins (POST vs. PRE ratio) yielded a condition-clustered heatmap (Figure 2b), delineating two divergent molecular signatures driven by the most significant differentially abundant proteins. As shown, proteins involved in cellular stress handling, proteostasis and trafficking are more abundant in the PRE condition. Among these proteins we found components of the COP9 multi-protein complex (COP9 signalosome, a central regulator of intracellular signaling controlling protein turnover) such as COPS5 (regulator of ubiquitin-dependent signaling), endosomal sorting (SNX1), and protein/RNA homeostasis factors (e.g., ERH, ARHGAP21). This pattern is consistent with a reduced requirement, after treatment, for stress-adaptive and turnover pathways that are typically engaged under inflammatory/oxidative pressure.

Conversely, in the POST condition, we found upregulated proteins whose molecular functions converge on flagellar motility, redox homeostasis, mitochondrial metabolism and membrane remodeling, all processes that are mechanistically aligned with improved sperm function. In particular, among the motility-related proteins, we identified TSSK4, a testis-specific serine/threonine kinase previously linked to sperm functions and motility-related phenotypes, and TBCE, a tubulin-folding cofactor involved in microtubule assembly that may impact axonemal organization and, consequently, flagellar structure. Moreover, several prominent drivers map to antioxidant/NADPH-related and mitochondrial pathways, including mitochondrial antioxidant enzyme PRDX3, and MPST (sulfur-transferase activity implicated in cellular redox control). In parallel, proteins linked to mitochondrial electron transport (e.g., UQCRH) and to lipid metabolism/membrane dynamics (e.g., ACSL3, which catalyzes acyl-CoA formation for lipid remodeling) emerged among the strongest contributors. Additional proteins in the POST-enriched cluster included factors involved in protein folding quality control, such as DNAJB4 (chaperone/co-chaperone function) and LAMP2 (lysosome/autophagy-related membrane protein), supporting a coordinated post-treatment remodeling of sperm cell homeostasis.

Overall, functional annotation of the most significant heatmap leaders indicates that Deprox-HP is associated with a structured shift in the sperm proteome, spanning redox/mitochondrial functions, membrane lipid handling, and proteostasis/trafficking pathways—providing a molecular framework consistent with the treatment-associated reduction in oxidative damage and the improvement in motility observed at the semen level.

### 2.3. Gene Ontology (GO) Enrichment of Differentially Expressed Sperm Proteins After Deprox-HP Treatment

**Figure 3:**
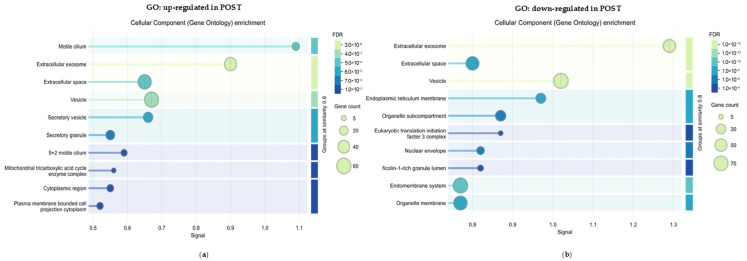
**Cellular compartment shifts associated with Deprox-HP treatment revealed by GO analysis.** Gene Ontology (GO) enrichment for the Cellular Component category was performed on proteins significantly modulated after treatment (POST vs. PRE; fold change > 1.5; FDR < 0.02). (**a**) Enriched GO terms for proteins upregulated in POST samples. (**b**) Enriched GO terms for proteins downregulated in POST samples. Bubble size reflects gene count, and color indicates FDR.

Gene Ontology enrichment analysis was performed using all significantly modulated proteins and revealed distinct functional pathways for the POST-dysregulated sets (up-and downregulated, respectively, Figure 3a,b).

Among POST-upregulated proteins, the most enriched cellular component terms were dominated by flagellar/motility structures (“motile cilium”, “9 + 2 motile cilium”), indicating increased representation of proteins supporting axonemal architecture and movement. This pattern is consistent with the functional endpoint observed at semen analysis, where sperm motility improved after treatment (Figure 1a), supporting convergence between molecular and conventional readouts. In the same upregulated set, enrichment of the mitochondrial tricarboxylic acid (TCA) cycle enzyme complex suggests a parallel reinforcement of mitochondrial bioenergetics.

In contrast, POST-downregulated proteins showed enrichment for endomembrane-associated compartments (endomembrane system, organelle membrane, endoplasmic reticulum membrane) together with intracellular localizations such as organelle subcompartment, nuclear envelope, and the eukaryotic translation initiation factor 3 (eIF3) complex. Overall, this profile points to a reduced representation of proteins linked to membrane trafficking/secretory machinery and translational hubs that are often engaged under cellular stress and inflammatory pressure, coherently aligning with the biochemical evidence of attenuated oxidative damage in the post-treatment condition (Figure 1b).

Notably, broad extracellular and vesicle-related terms (extracellular exosome, extracellular space, vesicle) emerged in both up- and downregulated sets. This bidirectional enrichment probably reflects modulation of distinct vesicle/exosome protein subsets—i.e., a qualitative shift in EV/vesicular cargo composition.

Taken together, these GO patterns underline a proteomic reinforcement of the motility apparatus and mitochondrial bioenergetic pathways, in line with the observed improvement of sperm movement parameters.

### 2.4. Pregnancy Cases: Longitudinal Semen/Oxidative Stress Profiling and Sperm Proteomic Remodeling

Two patients (P7 and P14) achieved a pregnancy during the 3-month Deprox-HP treatment period. One of the two pregnancies was achieved after 2 months of treatment, whereas the other occurred at the end of the treatment period (3 months). Moreover, one pregnancy resulted in a full-term delivery, while the other is currently ongoing at 19 weeks and is progressing physiologically.

As shown in Table 1, sperm lipid peroxidation (LP_sperm) decreased from PRE to POST in both cases (P7: 545 to 322 µEq/L; P14: 640 to 400 µEq/L). Semen parameters displayed patient-specific trajectories. P14 showed improvements in motility (total: 66 to 75%; progressive A + B: 35 to 47%) and sperm morphology (% typical forms: 6 to 9%), with sperm concentration remaining stable (250 to 260 ×10^6^/mL). In P7, sperm concentration did not improve over the treatment period, while sperm motility showed a slight increase (total: 58 to 63%; progressive A + B: 28 to 33%); however, sperm morphology increased (% typical forms: 5 to 10%) as well as sperm agglutination zones (S.A.), a finding commonly associated with inflammatory seminal features [14], resolved from PRE to POST, from 2nd grade to complete absence (0).

**Figure 4:**
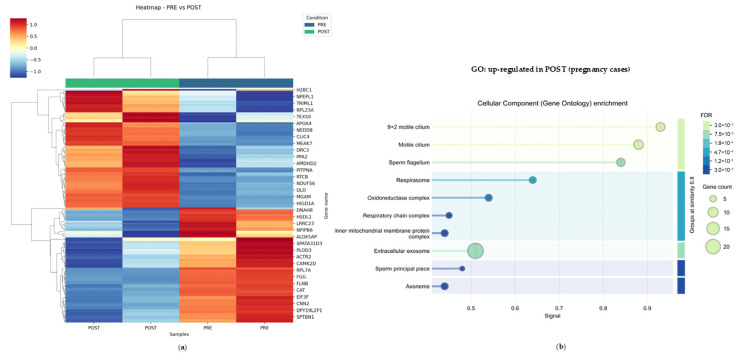
**Pregnancy-associated sperm proteomic signature after Deprox-HP treatment.** (**a**) Hierarchical clustering heatmap of the most significantly differentially abundant sperm proteins in the two patients who achieved pregnancy (P7 and P14), showing separation of POST from PRE samples based on scaled relative abundance (rows: proteins; columns: samples). (**b**) STRING-based Gene Ontology enrichment (Cellular Component) of proteins upregulated in POST samples from the pregnancy cases. Bubble size indicates gene count and color denotes FDR.

Proteomic analysis of purified spermatozoa from these two cases identified a robust POST/PRE separation with POST segregating from PRE samples in the heatmap of the most significant differentially expressed proteins (Figure 4a). All the identified proteins in P7 and P14 samples are listed in Supplementary File S3. Of note, we found TEX50, a testis-enriched sperm membrane protein that has been shown to be essential for male fertility [15], among the proteins most significantly enriched after treatment—an effect that was not detected in the global POST/PRE analysis across the entire cohort. STRING-based GO enrichment highlighted, as reported also above in Figure 3a, cellular component terms related to flagellar architecture and motility (“9 + 2 motile cilium”, “motile cilium”, “sperm flagellum”, “axoneme”, “sperm principal piece”), alongside mitochondrial respiratory/redox-associated compartments (“respirasome”, “respiratory chain complex”, “oxidoreductase complex”, “inner mitochondrial membrane protein complex”) (Figure 4b). These pathways outline a coherent post-treatment signature centered on motility apparatus integrity and mitochondrial bioenergetics/redox balance; all features are plausibly supportive of improved sperm functional competence in these pregnancy-associated responders. Overall, in these pregnancy cases, the clinical course was accompanied by a consistent reduction in sperm lipid peroxidation and a treatment-associated sperm proteomic shift toward flagellar/ciliary and mitochondrial respiratory compartments—molecular signatures aligned with sperm functions—supporting concordance between oxidative stress attenuation and sperm proteome remodeling during Deprox-HP administration.

## 3. Discussion

This study combines conventional semen analysis, oxidative stress profiling, and sperm proteomics to evaluate a 3-month Graminex™-based nutraceutical intervention (Deprox-HP) in patients diagnosed with MAGI. MAGI is clinically heterogeneous and can compromise fertility through altered glandular secretions and seminal plasma composition, partial obstruction, direct sperm injury, and inflammation-driven oxidative stress [4,16]. Within this landscape, we enrolled patients diagnosed with infertility-associated MAGI; our data converge on a motility-centered functional improvement upon nutraceutical supplementation accompanied by a marked reduction in sperm lipid peroxidation, together with coordinated PRE–POST remodeling of the sperm proteome involving mitochondrial/redox and membrane-associated pathways. Because Deprox-HP is primarily intended to modulate the inflammatory/redox milieu, we expected its main effects to emerge on functional sperm endpoints (e.g., motility) and oxidative damage rather than on spermatogenic output. Accordingly, sperm concentration did not exhibit significant changes over the study period. Motility is a key determinant of sperm transport through the female reproductive tract and is commonly associated with male fertility potential. Consistently, GO enrichment analysis indicated that the post-treatment proteomic shift preferentially involved flagellar/ciliary and mitochondrial bioenergetic compartments, providing a molecular framework for the observed motility improvement. In parallel, the two pregnancy cases provide an exploratory signal of molecular response, including selective TEX50 enrichment, consistent with heterogeneity in treatment-associated trajectories.

The significant increase in total and progressive motility alongside the marked decline in sperm lipid peroxidation is mechanistically concordant. Because sperm membranes are rich in polyunsaturated fatty acids and mature sperm have limited cytoplasmic antioxidant reserves, excessive ROS can drive lipid peroxidation, reduce membrane fluidity, and impair motility as well as key fertilization-related processes (capacitation signaling, acrosome reaction, sperm–oocyte interaction) [7,9,17]; therefore, a decrease in lipid peroxidation can impact positively on fertilization potential of sperm cells. In inflammatory genital tract conditions, leukocyte-derived ROS and a cytokine-rich milieu further amplify this shift from physiological ROS signaling to pathological oxidative burden [10,16,18]. Notably, despite only a variable upward trend in the seminal antioxidant capacity, lipid peroxidation decreased robustly, consistent with reduced net oxidative membrane injury (lower ROS generation and/or enhanced local detoxification) without a uniform rise in bulk seminal antioxidant capacity [9,18]. Clinically, this convergence supports lipid peroxidation as a meaningful intermediate endpoint to monitor MAGI, particularly in abacterial or persistent inflammatory phenotypes where antibiotics are not indicated [4,9,18,19].

Proteomics adds mechanistic support to the clinical findings. Unsupervised multivariate analysis showed a clear PRE–POST separation, indicating that the dominant proteomic variance tracks treatment status rather than random fluctuation. Given that mature sperm are transcriptionally quiescent, this remodeling likely reflects changes during epididymal transit and/or altered seminal plasma conditions (including EV-mediated cargo transfer), together with a shift toward less-damaged sperm subfractions under reduced oxidative pressure [20,21,22]. Accordingly, the proteomic shift is consistent with a “microenvironmental reset” rather than de novo protein synthesis. At the pathway level, POST samples were enriched for mitochondrial/redox proteins (e.g., PRDX3, MPST) and electron transport components (e.g., UQCRH), supporting reinforcement of mitochondrial redox homeostasis—a key determinant of sperm ROS balance and motility-related energetics in the midpiece [5,9]. Concurrent enrichment of proteins involved in lipid handling and membrane dynamics (e.g., ACSL3) is also coherent, as physiological lipid remodeling is required for maturation and capacitation and is readily disrupted by oxidative stress via lipid peroxidation and maladaptive lipid signaling [7,17,23]. The presence of proteostasis/quality-control factors (e.g., DNAJB4, LAMP2) further supports coordinated post-treatment homeostatic remodeling, whereas PRE-enriched ubiquitin/trafficking proteins (e.g., COP9 signalosome components, sorting nexins) are consistent with a stress-adaptive, damage-handling profile under inflammatory/oxidative load [9,23]. GO enrichment further refines this interpretation by indicating that the post-treatment remodeling also maps to compartments directly relevant to movement and energy supply. Of note, POST-upregulated proteins were dominated by flagellar/ciliary structures (“motile cilium”, “9 + 2 motile cilium”), consistent with increased representation of proteins supporting axonemal architecture and movement and offering a coherent mechanistic link with the improvement in motility observed at semen analysis. In addition, enrichment of the mitochondrial tricarboxylic acid (TCA) cycle enzyme complex suggests a coordinated reinforcement of mitochondrial bioenergetics. In the context of reduced oxidative damage, this pattern is compatible with a partial recovery of metabolic homeostasis with improved efficiency of oxidative metabolism and ATP-generating capacity required to sustain flagellar beating; indeed, even though glycolysis alone can sustain sperm viability, oxidative phosphorylation is necessary for proper sperm differentiation and maturation [24]. Conversely, the POST-downregulated set was enriched for endomembrane-associated compartments (endomembrane system, organelle membrane, endoplasmic reticulum membrane) together with intracellular localizations and assemblies such as the organelle subcompartment, nuclear envelope, and the eukaryotic translation initiation factor 3 (eIF3) complex. Although mature sperm have limited canonical ER/translation machinery, the presence of these annotations in the downregulated proteome likely captures a reduced representation of membrane-trafficking/secretory and translational hub-associated proteins that are often engaged under cellular stress and inflammatory pressure, coherently aligning with the biochemical evidence of attenuated oxidative damage after treatment. Moreover, EV-related cellular component terms (extracellular exosome, extracellular space, vesicle) appear in both the up- and downregulated sets; high-level GO categories can capture different EV/vesicular cargo modules, supporting a qualitative shift in vesicle/exosome composition (and possibly sperm–EV interactions) rather than a mere change in EV abundance. Within this framework, Deprox-HP may dampen stress-associated vesicle/endomembrane programs while favoring vesicle-associated proteins that accompany improved sperm structural integrity and energetic competence. Direct seminal EV profiling (particle counts, cargo proteomics/miRNA, functional assays) will be required to distinguish changes in EV abundance from cargo composition or sperm–EV interactions [20,21,22,25,26,27].

Existing evidence provides biological plausibility for anti-inflammatory/antioxidant actions of pollen-derived preparations and related phenolic constituents. Graminex™ pollen preparations have been characterized for their phenolic profile and reported protective effects in experimental contexts [28], while clinical studies of pollen–extract-based combinations have reported benefits on lower urinary tract symptoms in patients with benign prostatic hypertrophy [29]. Carvacrol, often discussed among relevant phenolic metabolites, has reported antioxidant and anti-inflammatory actions and can modulate NF-κB signaling in multiple experimental systems [13].

The selective TEX50 enrichment in these two cases is notable for two reasons. Although TEX50 was detected in the overall cohort, it did not emerge as significantly enriched in the global POST vs. PRE comparison. In contrast, when the analysis was restricted to the two patients who achieved pregnancy, TEX50 was among the most significantly increased proteins, showing a concordant PRE–POST rise in both individuals. Interestingly, TEX50 has been shown in mice to be essential for male fertility, with a key role in preserving acrosome/head integrity during epididymal transit and preventing epididymal sperm malformations with a globozoospermia-like phenotype when disrupted [15]. This finding indicates that TEX50 could be a critical node of sperm maturation during epididymal transit [15,20]. In this context, the selective post-treatment increase in TEX50 in pregnancy-associated responders could be consistent with the hypothesis that Deprox-HP—possibly via attenuation of inflammatory/oxidative pressure—promotes a more physiologic maturation program, including improved stabilization of head/acrosomal structures during transit. We may cautiously speculate that this finding aligns with the selective post-Deprox improvement in sperm morphology observed in the two patients who achieved a pregnancy. If validated in a larger cohort, and by additional biochemical and molecular techniques, TEX50 may represent a candidate marker of restored maturation integrity, supporting the broader value of sperm proteomics for identifying clinically relevant molecular response patterns in MAGI [9,15,20].

## 4. Materials and Methods

### 4.1. Patients and Study Design

Twenty patients (age between 32 and 49 years) diagnosed with complicated MAGI who attended the Center for Natural Procreation and Infertility Care (ISI-CPNCI) at Policlinico A. Gemelli IRCCS for primary infertility (unprotected intercourse for >12 months) were enrolled in the study. Deprox-HP was administered orally every day to all patients for 3 months. Deprox-HP is a food supplement based on a flower pollen extract belonging to the Poaceae family (Graminex^®^ G60^®^, Graminex^®^ NAX™) (IDI Pharma, Catania, Italy). This extract contains multiple substances that are useful for the body’s well-being: 21 amino acids, enzymes, coenzymes, minerals, carotenoids, flavonoids, phenols (including carvacrol), fatty acids (including alpha-linolenic acid), trace elements, and water- and fat-soluble vitamins. This study was conducted in accordance with the declaration of Helsinki, as revised in 2013, and all subjects provided written, informed consent. Approval for sperm collection and analysis was obtained from the Fondazione Policlinico Universitario A. Gemelli, IRCCS, local ethics committee (protocol ID5878, date of approval: 22 February 2024). The inclusion criteria were as follows: age between 20 and 50 years; clinically and ultrasonographically confirmed diagnosis of MAGI; and written informed consent to participate in this study. The exclusion criteria were as follows: positive semen culture for bacterial or fungal infections requiring targeted antibiotic therapy; presence of symptomatic varicocele and/or varicocele grade > I according to Dubin–Amelar classification; previous pelvic surgery or radiotherapy, or history of prostate and/or testicular cancer; history of cryptorchidism; orchitis; testicular trauma or torsion; CFTR (Cystic Fibrosis Transmembrane Conductance Regulator) gene mutations; and fever or antibiotic use within the three weeks preceding enrollment.

### 4.2. Semen Analysis

Semen samples were collected after 3–5 days of sexual abstinence. Following liquefaction for 30 min at 37 °C, semen analysis was performed according to the WHO 2021 guidelines [30]. Semen evaluation was independently conducted by at least two trained operators. The evaluation included various features, including pH, volume (mL), sperm count (10^6^/mL), total sperm count (10^6^/ejaculate), total and progressive A + B motility (%), morphology (% of normal and abnormal forms), and leukocyte count (10^6^/mL). In particular, motility A + B refers to progressive sperm motility, related to the sum of rapidly progressive (A) and slowly progressive (B) spermatozoa, as commonly reported in routine semen analysis. Sperm agglutination (S.A.) was assessed on fresh semen and graded according to the WHO manual [30] on a 0–4 scale, based on the extent of motile spermatozoa involved and the size of agglutinated clusters, from absent/isolated small clumps (0) to extensive/near-complete agglutination (4th grade). After liquefaction, spermatozoa were purified from other non-nemaspermic components by layering semen on a 50% Percoll^TM^ solution (GE Healthcare, Uppsala, Sweden) density gradient prepared with HEPES-buffered Ham’s F10 medium (Gibco, Life Technologies, Paisley, UK) containing sodium bicarbonate (0.2% NaHCO_3_; *w*/*v*; Merck, Darmstadt, Germany). The samples were then centrifuged at RT for 30 min at 400× *g*. The resulting pellet of purified sperm was collected, washed in PBS, and finally lysed in 2% SDS for proteomic analysis.

### 4.3. Reactive Oxygen Species (ROS) Assessment

Reactive oxygen species were evaluated before and after 3 months of Deprox-HP supplementation in seminal plasma freshly obtained after seminal fluid centrifugation (3000× *g* for 10 min) through a dual assessment of seminal plasma antioxidant capacity and sperm membrane lipid peroxidation. Antioxidant capacity was assessed using the anti-OX sperm kit (Diacron International Srl, Grosseto, Italy), which exploits the ability of antioxidants to reduce ferric iron (Fe^3+^) to ferrous iron (Fe^2+^). The reduced iron reacts with a specific chromogenic reagent, producing a red–violet color. Color intensity, measured spectrophotometrically, is directly proportional to the concentration of antioxidant molecules in the sample. Lipid peroxidation was evaluated using the LP-sperm kit (Diacron International Srl, Grosseto, Italy), which measures hydroperoxides derived from the peroxidation of both saturated and unsaturated lipids. The method is based on the ability of peroxides to oxidize ferrous iron to ferric iron, which then binds to the chromogenic mixture, generating a colored compound measurable photometrically. Quantification was performed by reading absorbance at 505 nm (Multiskan SkyHigh Microplate Spectrophotometer, ThermoFischer, Waltham, MA, USA), and the results are expressed as µEq/L according to the manufacturer’s protocol. The increase in absorbance is directly proportional to the concentration of lipoperoxides in the sample.

### 4.4. Proteomic Analysis

Proteomic analysis was performed using a bottom-up approach following enzymatic digestion with trypsin, a protease that specifically cleaves peptide bonds at the carboxyl side of lysine and arginine residues. Samples obtained from 15 patients (each before and after Deprox-HP supplementation) were prepared using the Filter-Aided Sample Preparation (FASP) protocol, which includes reduction, alkylation, and digestion steps. Centrifugal filters were conditioned with 100 μL of 1% (*v*/*v*) formic acid (FA) and centrifuged at 11,000× *g* for 15 min. Subsequently, 50 μg of samples were loaded onto the filters and centrifuged under the same conditions. Filters were washed with 200 μL of urea buffer (8 M urea, 100 mM Tris) and centrifuged at 13,000× *g* for approximately 20 min. Protein reduction was achieved by adding 100 μL of freshly prepared 8 mM dithiothreitol (DTT) in urea buffer, followed by incubation at 56 °C for 15 min and centrifugation at 13,000× *g* for 10 min. Excess DTT was removed by washing with urea buffer and centrifugation. Alkylation was performed by adding 100 μL of 50 mM iodoacetamide (IAA) in urea buffer, freshly prepared due to light sensitivity, followed by incubation at 37 °C for 20 min and centrifugation. Residual IAA was removed by washing with urea buffer and subsequent reduction with DTT. Buffer exchange was performed using 50 mM ammonium bicarbonate (AMBIC). Filters were transferred to new tubes, and digestion was initiated by adding 50 μL of trypsin prepared in AMBIC at an enzyme-to-protein ratio of 1:50. Digestion was carried out for approximately 16 h at 37 °C in a humidified chamber. Peptides were collected by centrifugation, acidified with trifluoroacetic acid (TFA) to a final concentration of 0.2%, evaporated using a SpeedVac, and stored at −80 °C. Peptide separation was performed using an UltiMate 3000 RSLC nano-HPLC System (ThermoFisher Scientific, Waltham, MA, USA) coupled with a high-resolution Orbitrap Fusion Lumos Tribrid Mass Spectrometer (ThermoFisher Scientific, Waltham, MA, USA) equipped with a nano-ESI source. Peptides were separated on a PepMap RSLC C18 column (2 µM, 100 Å, 50 µm × 15 cm, ThermoFisher Scientific, Waltham, MA, USA) using gradient elution. Eluent A consisted of an aqueous solution of 0.1% FA (formic acid), while eluent B was ACN with 0.1% FA. The gradient program was as follows (total runtime: 155 min): 3% B and 97% A (min 0–110), 20% B and 80% A (min 110–120), 40% B and 60% A (min 120–125), 90% B and 10% A (min 125–145), and 3% B and 97% A (min 145–155), with a flow rate of 0.3 μL/min. Each injection volume was 5 μL (containing a total of 1 μg of peptides), in positive polarity (voltage 1800 V). MS parameters included data-dependent scan mode (DDS), allowing automatic selection and fragmentation of the most abundant ions for acquiring high-resolution MS/MS spectra with an Orbitrap detector, with a resolution of 120,000 in the 375–1500 *m*/*z* range, and HCD fragmentation. Raw data were processed for protein identification and relative quantification, followed by chemometric analysis.

### 4.5. Bioinformatic Analysis

Raw spectra were processed using Proteome Discoverer software version 3.2, employing the SEQUEST-HT algorithm for protein identification against the UniProtKB/Swiss-Prot *Homo sapiens* database. Data processing consisted of a “processing” phase for peptide reconstruction and a “consensus” phase for protein assembly. Validation parameters included a false discovery rate (FDR) of 0.01 (strict target) and 0.05 (relaxed target). High-confidence peptide identifications were retained. Functional interaction and pathway analyses were conducted using STRING software (Version 12.0) to investigate protein localization and involvement in biological pathways. The proteomic dataset was initially filtered in high confidence to retain proteins identified with at least two unique peptides. Subsequently, data processing and imputation were performed using Omicscope version 1.5.0 (https://omicscope.ib.unicamp.br/OmicScope, accessed on 20 January 2026), with the following parameters: exclusion of contaminants was enabled; missing values were imputed using the mean; data were average-normalized and log-transformed; fold change cutoff was set at 1.5; and statistical analysis was performed using a paired *t*-test with *p*-value adjusted for multiple comparison according to the Benjamini–Hochberg procedure (adjusted *p*-value cutoff of 0.05) for the overall analysis, whereas row *p*-values were reported for the pregnancy cases analysis. Differential expression analyses were carried out comparing POST vs. PRE ratio. Volcano plot and heatmap visualization were also generated using Omicscope. Functional interaction and pathway analyses were conducted using STRING software to investigate protein localization and involvement in biological pathways.

### 4.6. Statistical Analysis

Data are reported as mean ± standard deviation (SD). Depending on the dataset, statistical comparisons were performed using a paired two-tailed Student’s *t*-test, a one-way ANOVA (for seminal parameters), or a one-sample *t*-test. The one-sample *t*-test was used when the control-group mean was normalized and set to 1. A *p* value < 0.05 was considered statistically significant. All analyses were carried out using GraphPad Prism v10.3.1.

In this paper, we made use of AI (ChatGPT, Version GPT-5.2 Thinking) to generate some sentences in the text and to edit the overall English language.

## 5. Conclusions

Taken together, our findings indicate that 3-month Deprox-HP administration in MAGI patients is associated with a clinically meaningful improvement in sperm motility and a marked reduction in sperm lipid peroxidation, accompanied by a coordinated sperm proteomic remodeling toward mitochondrial/redox homeostasis, membrane remodeling, and flagellar/motility-related compartments. These convergent functional and molecular readouts support Deprox-HP as a potentially valuable adjunct therapeutic option, particularly in inflammatory phenotypes where antibiotics are not indicated and management is largely supportive. Moreover, these findings suggest that Deprox-HP may also be worth evaluating in other male infertility settings characterized by inflammatory/oxidative dysregulation (e.g., idiopathic oxidative stress, varicocele-associated dysfunction, or chronic prostatitis-like syndromes), pending confirmation in larger controlled studies. Indeed, the main limitations of the present work reside in the absence of a control group (e.g., placebo or untreated controls) and in the relatively small sample size, reflecting its pilot design; therefore, these observations warrant confirmation in larger, controlled cohorts with predefined reproductive endpoints. Ultimately, this study supports the concept that integrating oxidative stress metrics with sperm proteomics can provide a mechanistically informed framework to monitor and stratify treatment response in inflammatory male infertility.

## Figures and Tables

**Table 1:** Semen parameters comparison between PRE and POST Deprox-HP in two patients who achieved pregnancy during the treatment period.

Patients	Sperm Count (×10^6^/mL)	Motility (% Total)	Motility (% A + B)	Morphology (% Typical Forms)	S.A.	LP_Sperm (µEq/L)
**P7_PRE**	170	58	28	5	presence, 2nd grade	545
**P7_POST**	105	63	33	10	absence, 0 grade	322
**P14_PRE**	250	66	35	6	absence, 0 grade	640
**P14_POST**	260	75	47	9	absence, 0 grade	400

Comparison of conventional semen parameters and oxidative stress readouts in patients P7 and P14 assessed at baseline (PRE) and after 3 months of Deprox-HP administration (POST), including sperm concentration, total and progressive motility, morphologically normal forms, sperm lipid peroxidation (LP_sperm), and the presence/grade of sperm agglutination (S.A., evaluated according to WHO criteria). Values are reported as measured at each time point.

## Data Availability

The original contributions presented in this study are included in the article and Supplementary Materials. Further inquiries can be directed to the corresponding author.

## Supplementary Materials

The following supporting information can be downloaded at: https://www.mdpi.com/article/10.3390/ijms27052498/s1.
